# Sirtuin1 expression and survival in endometrial and clear-cell uterine cancer

**DOI:** 10.1007/s00418-020-01873-x

**Published:** 2020-05-09

**Authors:** Susanne Beyer, Fangfang Chen, Sarah Meister, Bastian Czogalla, Theresa M. Kolben, Anna Hester, Alexander Burges, Fabian Trillsch, Elisa Schmöckel, Doris Mayr, Artur Mayerhofer, Sven Mahner, Udo Jeschke, Thomas Kolben

**Affiliations:** 1 Department of Obstetrics and Gynecology, University Hospital, LMU Munich, Marchioninistr. 15, 81377 Munich, Germany; 2 Institute of Pathology, University Hospital, LMU Munich, Marchioninistr. 15, 81377 Munich, Germany; 3 Biomedical Center Munich (BMC), Cell Biology, Anatomy III, LMU Munich, Großhadernerstraße 9 Martinsried, 82152 Planegg, Germany

**Keywords:** Sirtuin1, HDAC, Endometrial cancer, Survival

## Abstract

Several risk factors like obesity and hyperlipidemia were described for endometrial cancer. Here, the nuclear NAD-dependent histone-deacetylase Sirtuin1 (SIRT1) seems to be important. SIRT1 is also involved in cell regulatory mechanisms and can serve as tumor promotor or suppressor. Its role in tumor biology is not clear yet. In this study, we evaluated and correlated the SIRT1 expression with patients’ tumor characteristics in endometrioid and clear-cell cancer of the uterus. 65 paraffin-embedded samples of patients with endometrial and clear-cell cancer of the uterus were immunohistochemically stained and SIRT1 expression was evaluated by immunoreactive score. The results were correlated to clinical and pathological tumor characteristics as well as to the expression of ARID1A and β-Catenin. The staining was significantly more intensive in uterine endometrioid carcinoma compared to uterine clear-cell carcinoma (*p* = 0.007). The expression of SIRT1 correlated significantly with the membranous expression of β-Catenin (*p* = 0.028) and ARID1A (*p* = 0.021). Patients with positive Sirtuin1 expression had a significantly better progression-free survival (*p* = 0.042), the overall survival showed a trend towards a better prognosis (*p* = 0.070). SIRT1 expression seems to be associated with improved progression-free survival in uterine cancer (endometrioid and clear-cell) and is correlated to the tumor suppressors β-Catenin and ARID1A. Further studies are necessary to elucidate the role of SIRT1 in uterine and ovarian cancer and its potential as a therapeutic target.

## Introduction

Endometrial cancer is the most common gynecologic cancer in developed countries (Siegel et al. 2018). The incidence in Germany is 11,700 (new diseases per year), which makes it the fourth common cancer among women in Germany [Robert Koch-Institut (Hrsg.) (2010)]. Traditionally, subtypes are divided into class I– estrogen dependent (low grade endometrioid cancers) and class II (estrogen-independent high-grade serous or clear-cell carcinomas) (Kurman RJ 2014). Several risk factors like obesity, hyperlipidemia, infertility or late onset of the menopause were described (Ali 2014; Bokhman 1983). Another risk factor seems to be the history of endometriosis (Zucchetto et al. 2009). Different mutations, like ARID1A and β-Catenin are common in endometriosis and associated cancers, where they can act as tumor suppressors (Wiegand et al. 2010).

Current cancer research focuses on epigenetic alterations. These can be caused by histone deacetylases (HDAC), for example Sirtuins 1–7. Sirtuin1 (SIRT1) belongs to the NAD + -dependent class III HDAC and is located in the nucleus (Banks et al. 2008; Michishita et al. 2005). Like other molecules being responsible for epigenetic alterations, SIRT1 affects the cell-cycle via multiple pathways (Pinzone et al. 2013): besides the regulation of lipid and glucose metabolism (Brunet et al. 2004), it is also involved in the pathogenesis of cancer by deacetylation of different histones (Vaquero et al. 2004). SIRT1 downregulates the tumor suppressor p53 and Fox P3 by deacetylation (Chadha et al. 2019) and interaction with other proteins are described (Pinzone et al. 2013; Vaziri et al. 2001). By multiple interactions, SIRT1 can act either as tumor promotor or as tumor suppressor (Lin and Fang 2013). Nevertheless, experiments have shown that mice with overexpression of SIRT1 have lower cancer risk and less metabolic dysfunction (Herranz et al. 2010).

In endometrial cancer, its expression is described to be generally increased (Asaka et al. 2015; Bartosch et al. 2016) as well as in endothelial ovarian cancers (Jang et al. 2009). In ovarian cancer, SIRT1 overexpression was correlated with improved overall survival (Jang et al. 2009). In endometrial cancer, its role regarding tumor growth is not clear yet (De et al. 2018). Some studies showed an increased cisplatin resistance with lower survival rates (Asaka et al. 2015), while other studies described an inhibition of cancer progression by stimulation of SIRT1 (Deus et al. 2017).

This study aimed to determine the expression of SIRT1 in endometrioid and clear-cell carcinomas of the uterus and to analyze potential correlations to clinical pathological characteristics including survival.

## Materials and methods

### Patients and specimen

65 patients with endometrial or clear-cell cancer of the uterus, who had undergone surgery at the Department of Gynecology at Ludwig–Maximilians-University in Munich between 1990 and 2001, were included in this study. Inclusion criteria were surgery in our department due to endometrioid or clear cell cancer of the uterus and samples being available as well as histo-pathological data. Due to the low number, other subtypes were excluded. Besides these criteria, no pre-selection was performed. The distribution was as follows: 59 patients with endometrioid uterine carcinoma and six with clear cell uterine carcinoma. Clinical, pathological and survival data were provided by the tumor registry of Munich. Patients’ characteristics are shown in Table 1.

**Table 1:** Patients characteristics

	*n*	%
Age (average ± SD)	64.6 (± 10.4)	
Histopathology		
Endometrioid uterine carcinoma	59	90.8
Clear cell uterine carcinoma	6	9.2
Grading		
Grade 1	18	27.7
Grade 2	24	36.9
Grade 3	23	35.4
FIGO		
I	12	18.5
II	13	20
III	18	27.7
IV	22	33.8
Tumor size		
pT1	50	76.9
pT2	7	10.8
pT3	8	12.3
pT4	0	0
Patients with		
Diabetes	8	12.3
Hypertonus	16	24.6
Adipositas	23	35.3
None	18	27.7
Progression (over 177 months)		
None	51	78.5
At least one	11	16.9
Not available	3	4.6
Survival (over 177 months)		
Right censured	32	49.2
Died	32	49.2
Not available	1	1.5

*SD* standard deviation

### Ethics approval

All samples were originally collected during surgery procedure for histopathological diagnostics and no longer used for it. Patient data were completely anonymized. Authors were blinded for clinical information during the analyses. The study was performed conforming the Declaration of Helsinki and was approved by the local ethics committee of the LMU Munich (reference number 19-249).

### Immunohistochemistry

Tissue microarray paraffin blocks were cut at 2–3 µm and prepared by heat-treatment. This was followed by the assessment of the primary antibody with an incubation time of 60 min at room temperature (Anti-SIRT1-Antibody; polyclonal antibody; dilution 1:180; company: Atlas antibodies; order number: HPA006295; antibody validation by isotype control and system control). After detection of the primary antibody, chromogen was put on samples and a counter staining with hematoxylin took place. All samples were stained at the Department of Pathology, Ludwig–Maximilians-University, Munich.

The expression was finally analyzed by the Remmele immunoreactive score (IRS) in a blind process. The intensity of the staining was scored between 0 and 3 (0 = no intensity, 1 = low intensity, 2 = moderate intensity, 3 = high intensity) and multiplied with a score representing the percentage of stained cells (0 = 0%; 1 = 1–10%; 2 = 11–50%; 3 = 51–80%; 4 > 80%). SIRT1 was dichotomized into no expression and expression. In previous studies, the same collective was already stained immunohistochemically with antibodies against ARID1A (ARID1A/BAF250a Rabbit mAb; New England Biolabs GmbH; antibody validation by manufacturer) and β-Catenin (β-Catenin Mouse IgG-1; Roche, Ventana, ready to use; antibody validation by manufacturer) (Wu and Roberts 2013). All those staining’s were performed at the Department of Pathology, Ludwig-Maximilians-University, Munich. To control the staining of SIRT1, non-pathological samples of human tonsils were stained.

For analyzing the images the light microscope “Immunohistochemistry Type 307–148.001 512 686” by Leitz was used. The camera was produced by Fissler (IH-Camera 3CCD Colour Video Camera). For image acquisition, the software “Discuss Version 4,602,017-#233 (Carl C. Hilgers Technical Office) was used. Image bit depth: 24 mm; time and space resolution data: 760 + 574 pixel.

### Statistics

IBM SPSS Statistics version 23 (Armonk, NY, USA) was used for statistical analyses. To calculate bivariate correlations, Spearman’s-rank-correlation coefficient was employed. To compare independent groups, we used non-parametric tests (NPAR: Kruskal–Wallis test, Mann–Whitney *U* test). Survival times were shown by Kaplan–Meier estimates and calculated by log-rank-test. For improved clarity, these results are shown in years, while calculations were performed in months. For statistical significance *p* value had to be < 0.05.

## Results

### Expression of SIRT1

Non-pathological tissue microarrays (TMA) of tonsils samples were used to control the staining.

**Fig. 1:**
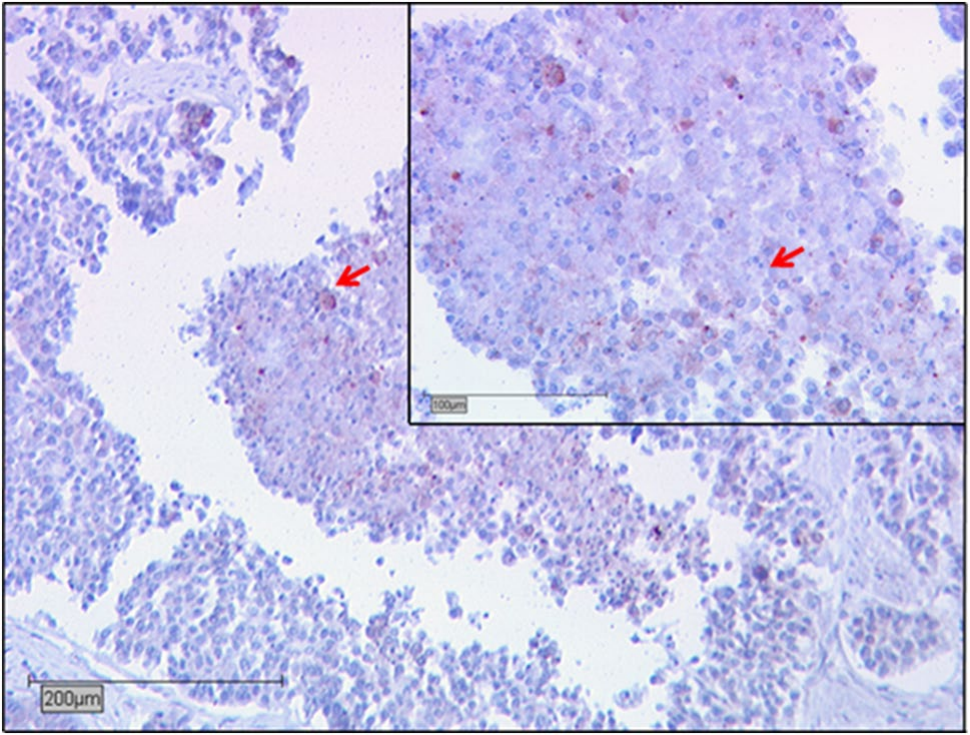
Sirtuin1 expression in endometrioid uterine carcinoma with an IRS score of 4: examples for Sirtuin1 positives cells are marked by →. Scale bar 200 µm, small pictures 100 µm

Regarding the whole sample, eight TMAs were not evaluable (12.3%) due to insufficient tissue quality. In our study group, SIRT1 was expressed in 35.4% of all samples with a median IRS score of 4 (SD: ± 2.89; Fig. 1). 16.9% of the evaluated samples did not show any expression at all.

**Fig. 2:**
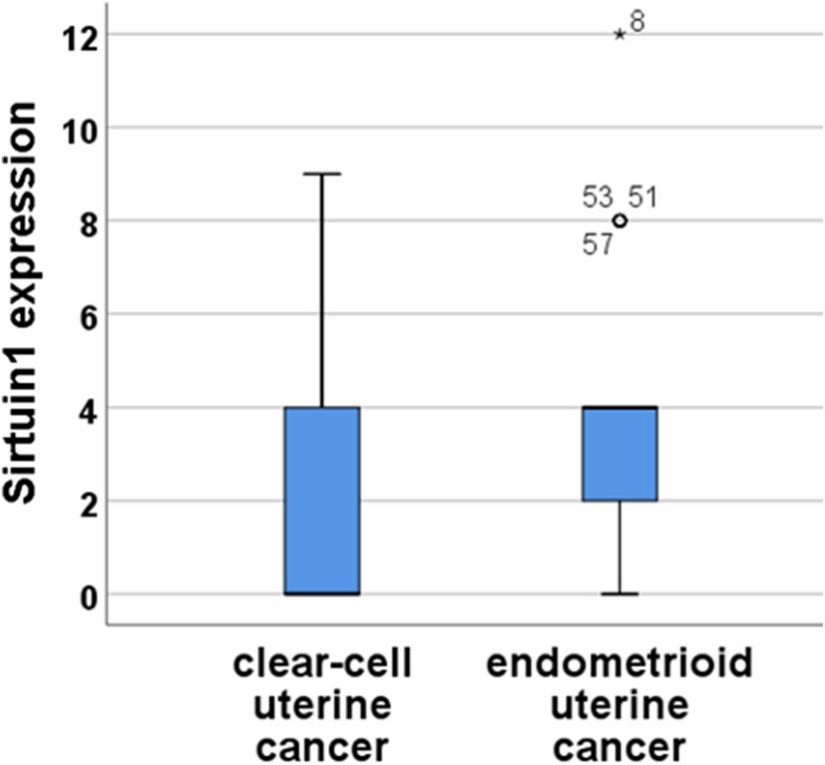
SIRT1 expression in clear-cell uterine (*n* = 6) and endometrioid uterine (*n* = 59) cancer shown in a boxplot. There is a significant higher IRS of the Sirt1 expression in endometrioid uterine cancer (IRS = 4) compared to clear-cell uterine cancer (IRS = 0); *p* = 0.007

The expression of SIRT1 was significantly higher in endometrioid carcinoma (median: 4; SD ± 2.66) compared to clear cell carcinoma (median: 0; SD ± 2.01; *p* = 0.007; Fig. 2; Table 2).

**Table 2:** Results of correlation between clinical pathologic characteristics including survival and expression of Sirtuin1

	Uterine cancer (*n* = 65)	
	Median (± SD)	*p*
Histology		**0.007**
Endometrioid	4 (± 2.66)	
Clear-cell	0 (± 2.01)	
T-stage (Gr.)		0.267
T1	4 (± 2.46)	
T2	4 (± 3.37)	
T3	6 (± 3.70)	
T4		
pN		0.572
pN0	4 (± 2.25)	
pN1	4 (± 2.64)	
FIGO		0.161
I	4 (± 1.94)	
II	4 (± 2.23)	
III	6 (± 3.44)	
IV	4 (± 2.58)	
Grading		0.760
G1	4 (± 2.72)	
G2	4 (± 3.01)	
G3	4 (± 2.63)	
Diabetes	–	0.858
Obesity	–	0.983
Hypertension	–	0.539
OS	–	**0.07**
PFS	–	**0.042**

*OS* overall survival, *PFS* progression-free survival, *KW* Kruskal–Wallis test

Significant results and important differences are shown in bold

### Correlation to pathological characteristics

No significant difference was detected regarding T-stage, FIGO-stage, grading and lymph-node status (pN) (Table 2). This was also the case when histological subtypes were analyzed. SIRT1 did not correlate to specific risk factors for endometrial carcinoma suh as diabetes (*p* = 0.858), obesity (*p* = 0.983) or hypertension (*p* = 0.539; Table 2).

### Correlation to survival

**Fig. 3:**
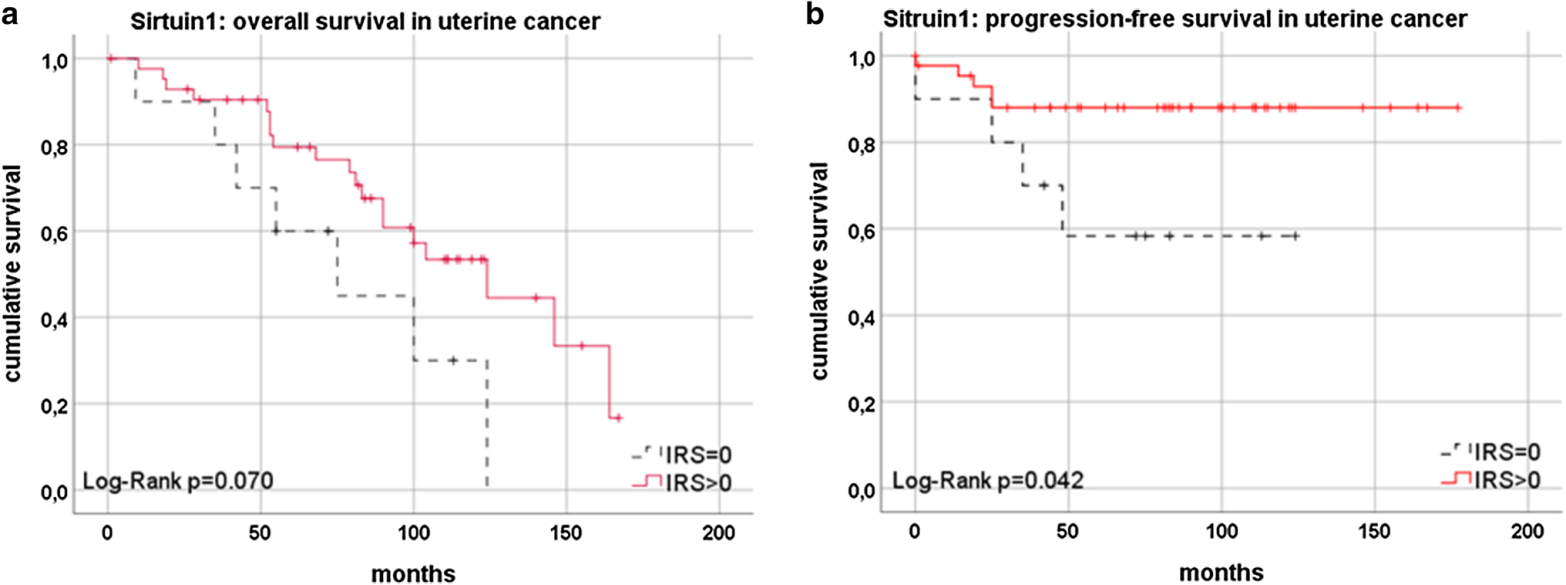
Survival in regard to SIRT1 expression [**a** trend to better overall survival in uterine cancer in case of SIRT1 expression (red) compared to missing SIRT1 expression (black) (*p* = 0.070); **b** significant better progression-free survival in uterine cancer in case of SIRT1 expression in uterine cancer (red) compared to missing SIRT1 expression (black) (*p* = 0.042)]

Patients with SIRT1 expression in endometrioid cancer of the uterus to show better overall-survival than patients without SIRT1 expression (*p* = 0.070; Fig. 3a; Table 2). Progression-free survival was significantly better in uterine endometrioid and clear-cell cancer for patients with SIRT1 expression (*p* = 0.042; Fig. 3b; Table 2).

### Correlation to other expression markers

The expression of SIRT1 in uterine and ovarian endometrioid and clear-cell carcinomas correlated significantly with a high expression of β-Catenin in the membrane (*p* = 0.028; *ρ* = 0.333, *p* = 0.011; Table 3). Additionally, the SIRT1 expression was positively correlated with high ARID1A expression but the correlation coefficient *ρ* was not significant (*p* = 0.021; *ρ* = 0.026, *p* = 0.850; Table 3).

**Table 3:** Correlation between SIRT1 and β-Catenin and ARID1A

	SIRT1	
	*p* (NPAR)	Correlation coefficient *ρ*
ARID1A	0.021	0.026 (*p* = 0.850)
β-Catenin (membranous)	0.028	0.333 (*p* = 0.011)

## Discussion

Endometrial cancer is classified into type-I (containing endometrioid types) and type-II cancers (including clear-cell carcinomas) (Kurman RJ 2014). Many studies exist observing the prognostic value of different epigenetic changes in uterine cancers, but less is known about the role of SIRT1 in these cancer types. The aim of the study was to evaluate the prognostic value of SIRT1 expression in endometrioid and clear-cell cancer of the uterus.

This histone-deacetylase is known to be involved in the pathophysiology of metabolic diseases and neurodegenerative disorders (Lavu et al. 2008). Regarding cancer progression its role is controversially discussed and is known to differ in respect to tissue and cancer entity: SIRT1 expression does not seem to have any prognostic significance in retinoblastoma (Batra et al. 2015). In contrast, in adenocarcinoma and small cell carcinoma of the lung, SIRT1 seems to be associated with poor survival, which can also be observed in large B-cell lymphoma and in clear cell renal cell carcinomas (Chen et al. 2017; Jang et al. 2008; Noh Baek et al. 2013a, b; Noh Kang et al. 2013a, b; Wang and Wang 2016).

In tumor cells of renal clear cell carcinoma SIRT1 was described to be associated with poor survival and was suggested to be a tumor promotor (Noh Kang et al. 2013a, b). We could not confirm that these finding can be generalized: SIRT1 expression in uterine clear-cell-carcinomas was not associated with poor survival rates in our study, but the number of clear-cell carcinomas was limited. This supports the assumption that the role of SIRT1 has to be examined specifically for each cancer entity and subtype.

Regarding gynecologic cancer entities, the role of SIRT1 is studied in cervical cancer. Here its knockdown seems to inhibit progression of paclitaxel-resistant cells (Xia and Zhou 2018) and its expression seems to predict the efficacy of neoadjuvant chemotherapy (Teramae et al. 2015). Until now, there are only few studies focusing on SIRT1 as prognostic factor in endometrial and clear cell carcinomas of the uterus. Up to now, the SIRT1 expression has only been described to be generally increased in endometrial cancer (Asaka et al. 2015; Bartosch et al. 2016) as well as in endothelial ovarian cancer types (Jang et al. 2009). In some studies, an increased cisplatin resistance with lower survival rates was observed (Asaka et al. 2015), while other studies showed that cancer progression was inhibited by stimulation of SIRT1 (Deus et al. 2017). Our results show that an increased SIRT1 expression in uterine cancer types (endometrioid and clear-cell) is correlated to a longer progression-free survival which supports the thesis that SIRT1 acts as a tumor suppressor. Studies already highlighted the role of the SIRT1-p53 pathway in this context (Gomes et al. 2016).

Also in regard to other gynecologic tumors, such as ovarian cancer, the role of SIRT1 expression is inconsistent: regarding the most common ovarian cancer types (serous, mucinous, endometrioid, clear-cell), an increased SIRT1 expression is significantly correlated to shorter survival rates (Mvunta et al. 2017). In contrast, Jang et al. showed in serous carcinomas that a decreased expression is correlated with decreased survival rates and high FIGO stages (Jang et al. 2009).

Heckel et al. found that the expression of ARID1A and absent nuclear β-Catenin staining were positive prognosticators in endometrioid and clear-cell carcinoma of the uterus and the ovaries (Heckl et al. 2018). We detected a positive correlation of SIRT1 expression in the examined entities with both proteins, ARID1A and β-Catenin. SIRT1 being a histon-deacetylase, one could hypothesize that its overexpression could lead to an activation of the transcription of the other proteins named above. The protein ARID1A is part of the SWI/SNF chromatin remodeling complex and is supposed to be a tumor suppressor (Wu and Roberts 2013). Expression of ARID1A as well as expression of SIRT1 correlated to better survival rates. Regarding β-Catenin, another tumor suppressor and part of the Wnt-pathway (Heckl et al. 2018), positive membranous and negative nuclear staining correlated to better survival rates, as well as SIRT1 expression. Both correlations support our thesis that SIRT1 functions as tumor suppressor in the examined uterine cancer types.

As in other histone deacetylases already tested, SIRT1 can be a target for individual therapy. Reservatol, a natural phenol, is known to stimulate SIRT1 (Deus et al. 2017). By this stimulation, an inhibition of cancer progression can be induced in vitro (breast cancer cells) (Deus et al. 2017). This approach could also be reasonable in uterine cancer.

A limitation of this analysis was the low number of specimen, especially of clear-cell carcinomas (6). Due to this fact, our findings can be seen as hypothesis and further studies with higher sample numbers would be useful and could probably help to confirm our thesis.

In conclusion, we detected a correlation of SIRT1 expression and β-Catenin and ARID1A in endometrioid and clear-cell cancer types of the uterus. Furthermore, progression-free survival was better in patients with SIRT1 overexpressing uterine tumors, while only a trend was observed in regard to overall survival. To our knowledge, this is the first analysis showing a correlation of SIRT1 and survival in uterine cancer. These results suggest that SIRT1 may act as tumor suppressor in this examined cancer entity. Further studies are necessary to elucidate the actual role of SIRT1 in endometrial cancer and its correlation with ARID1A an β-Catenin. SIRT1 may act as potential target in a tailored therapy approach. In this context, the potential of Reservatol should be further analyzed.

## Abbreviations

HDAC: Histone deacetylases
IRS: Immunoreactive score
KW: Kruskal–Wallis test
NPAR: Non-parametric tests
OS: Overall survival
PFS: Progression-free survival
SIRT1: Sirtuin1
TMA: Tissue microarray
